# The Role of MicroRNAs in Mitochondrial Homeostasis and their Involvement in the Pathogenesis of Obesity and Metabolic Syndrome: A Focus on MicroRNAs

**DOI:** 10.2174/0109298673361354250224060829

**Published:** 2025-03-07

**Authors:** Natalia Todosenko, Kristina Yurova, Maria Vulf, Olga Khaziakhmatova, Vladimir Malashchenko, Igor Khlusov, Alexandra Komar, Ivan Kozlov, Larisa Litvinova

**Affiliations:** 1 Center for Immunology and Cellular Biotechnology, Immanuel Kant Baltic Federal University, 236001, Kaliningrad, Russia;; 2 Laboratory of Cellular and Microfluidic Technologies, Siberian State Medical University, 2, Moskovskii Trakt, 634050, Tomsk, Russia;; 3 Institute of Professional Education, I.M. Sechenov First Moscow State Medical University, Moscow, Russia

**Keywords:** Mitochondria, metabolic syndrome, obesity, microRNAs, mitomicroRNAs, cell cultures

## Abstract

The maintenance of the functional potential of mitochondria is directly related to epigenetic factors, microRNAs (miRs), and mitomicroRNAs (mitomiRs). An important role in the development of metabolic syndrome (MetS)/obesity is attributed to miRs, which have pro-inflammatory or anti-inflammatory potential and can penetrate the mitochondrial matrix. Deciphering the mechanisms responsible for the transport of miRs into the mitochondria would, we believe, allow us to use the knowledge obtained to build designs for the transport of drugs/mitomiRs into cells/mitochondria with low toxicity. A thorough understanding of the polyfunctionality/versatility of individual mitomiRs in specific cells (cell cultures, tissues: adipocytes, brain cells) will allow targeting cellular metabolism to comprehensively block the central link in disease pathogenesis with low potential side effects of this treatment. In this review, we have attempted to identify the key miRs/mitomiRs associated with MetS that affect mitochondrial function. In our opinion, further research should focus specifically on the miR/mitomiRs described here and further investigate their potential in the development of MetS and its components.

## 1 INTRODUCTION

Metabolic syndrome (MetS) and its components are widespread pathologies of our time that have reached the scale of a global pandemic [1]. The theory of transgenerational inheritance may provide some explanations for the mechanism that leads to a growing proportion of the human population suffering from these diseases. Genetic changes associated with the development of MetS and obesity form the basis of a “memory” that is passed down through generations and may play the role of a leading factor in the development (predisposition to the development) of metabolic disorders at the level of the main regulatory center of eating behavior-the hypothalamus and other structures of the central nervous system [2].

Appetite is regulated homeostatically and hedonistically at the level of the hypothalamus, hypothalamus humoral signaling, hippocampus, amygdala, prefrontal cortex, and midbrain [3, 4]. Obesity alters the phases of appetite and increases the resistance of target neurons to hormonal influences at the blood-cerebrospinal fluid barrier, at the receptor level, and the intracellular level, leading to excessive food intake and changes in the processing of reward signals [5].

In the brain, against the background of obesity, mitochondrial dysfunction becomes a central event [6-8], leading to increased oxidative stress, inflammation, protein aggregation, changes in hormone levels, insulin resistance (IR), and disruption of the structural integrity of the blood-brain barrier [9, 10]. On the other hand, peripheral organs and systems also suffer energy loss due to mitochondrial dysfunction against the background of MetS [11]. Mitochondrial dysfunction is thus clearly involved in the pathogenesis of metabolic disorders, which makes the restoration of its functional potential an important task for genetics, molecular biology, and medicine.

Mitochondrial dysfunction is largely associated with the epigenetic component, the key element of which is microRNAs [12]. MicroRNAs (miRs) are endogenous non-coding RNAs that regulate gene expression [13]. At the same time, some miRs migrate into the mitochondrial matrix or target cytoplasmic messenger RNAs [14, 15] (mitomiRs) and regulate mitochondrial homeostasis at the gene level (including mtDNA).

mitomiRs is a new topic that has been under active investigation since 2010. Over the course of 14 years, only 88 publications with the keyword “mitomiRs” were found in the Pubmed database (searched May 23, 2024); mitomiRs is a new topic that has been actively developing since 2010. Over the course of 14 years, only 88 publications for the keyword “mitomiRs” and only 3 articles for the phrase “mitomiRs and obesity” were found in the Pubmed database (https://pubmed.ncbi.nlm.nih.gov/; retrieved on 23.05.2024). Metabolic syndrome modulates mitomiRs that control the activity of mitochondrial target genes in adipose-derived mesenchymal stem cells (MSCs) and impairs ATP molecule production [16]. The use of mitomiRs could be a potential new mitoprotective strategy to prevent the development of obesity, MetS, and related diseases and to preserve the regenerative potential of stem cells.

The aim of this review is to describe the role of miRs (mitomiRs) present in mitochondria and to identify their potential role in the regulation of mitochondrial functions in inflammatory processes associated with the development of obesity/MetS.

**Fig. (1):**
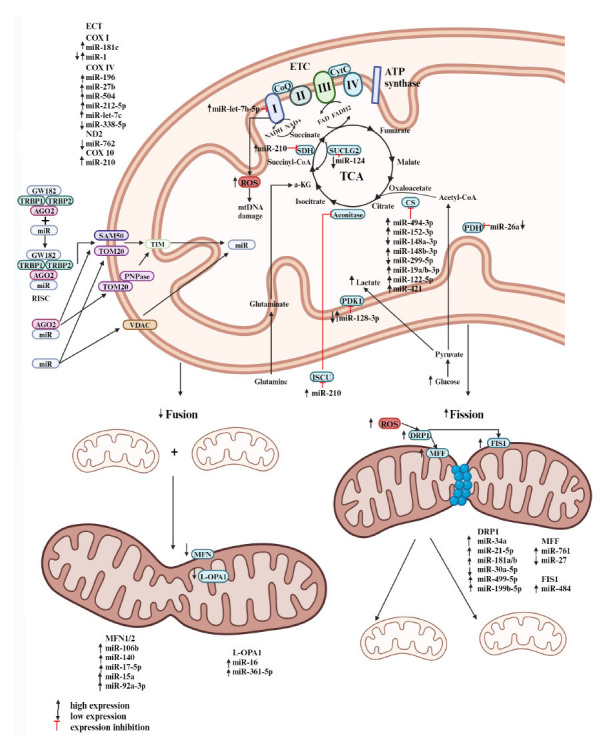
Involvement of microRNAs (miRNAs) in the regulation of metabolism and biogenesis (fission/fusion) of mitochondria in metabolic syndrome and its components (biorender.com).

MiRs are integrated into the RISC protein complex, interact with AGO2, or move freely into the mitochondrial matrix *via* transporters localized in the outer mitochondrial membrane (SAM50, TOM20, PNPase, VDAC) and the inner mitochondrialmembrane (TIM). MetS is associated with high production of ROS due to impaired function of the respiratory chain, electron transport chain, and tricarboxylic acid cycle (TCA) in the mitochondria. These alterations are associated with miRs that can enter the mitochondria (Fig. **1**) and exert their effect on mtDNA genes. In addition, metabolic syndrome is characterized by increased mitochondrial fission and decreased fusion, indicating a violation of organelle biogenesis. The review describes miRs/mitomiRs involved in the regulation of mitochondrial fusion/cleavage proteins in the context of MetS/obesity.

## 2 MITOCHONDRIA AND METABOLIC SYNDROME

### 2.1 Mitochondria and their Functions

Mitochondria are intracellular organelles (diameter 0.5-1 μm) with a double membrane that communicate in a complex way and form a network within the cells [17]. The outer mitochondrial membrane (OMM) is adjacent to the cytoplasm and contains a large number of proteins involved in the normal function of the organelle, its communication, and intracellular transport [17]. The OMM structure is porous due to the content of porin proteins and allows the transport of ions and small uncharged molecules into the intermembrane space [18]. The movement of larger proteins occurs with the participation of translocase proteins [18].

The inner mitochondrial membrane (IMM) is characterized by a tightly folded structure that provides a surface for electron transport chain (ETC) components and forms cristae.

The IMM allows molecules and proteins to access exclusively *via* membrane transport proteins that are selective for specific proteins and ions [18].

Ketogenesis, amino acid metabolism, the synthesis of steroid hormones, heme synthesis, and the tricarboxylic acid cycle take place within the mitochondria (in the matrix). The intermembrane space (IMS) is located between the OMM and the IMM [17].

The separation between the matrix and the IMS enables the mitochondria to produce ATP. The tightness of the IMM allows proteins to pump hydrogen ions from the matrix into the IMS, creating an electrochemical gradient that ATP synthase uses to produce ATP through the process of oxidative phosphorylation (OXPHOS). In addition, mitochondria regulate cell signaling and differentiation [18].

Mitochondrial metabolism involves biochemical reactions within the mitochondria and the interaction of the mitochondria with the cytosol [17].

The mitochondrial matrix contains the genome, ribosomes, and other structures [18]. Mitochondria have their own circular DNA (mtDNA), which is about 16 kb long and consists of light and heavy chains. The heavy chain of the mtDNA contains the majority of the coding sequences. The mitochondrial genome encodes 37 transcripts: 13 encode electron transport chain (ETC) protein subunits, there are 2 rRNAs (coding sequences for short peptides incorporated into the genes), and 22 mRNAs [17].

It is assumed that 99% of the more than 1000 mitochondrial proteins are encoded by the nuclear genome and synthesized in the cytoplasm [19], then these proteins are transferred into the mitochondria by transporter proteins: Outer membrane translocase - TOM and Inner membrane translocase - TIM [17]. The mitochondrial genome encodes the remaining 1% of mitochondrial proteins, and the mitochondrial ribosome is responsible for the translation of these mRNAs [19]. Mitochondria have been found to have their mechanism for protein synthesis [19].

### 2.2 The Involvement of Mitochondria in the Pathogenesis of Metabolic Syndrome

Mitochondrial dysfunction is an umbrella term that encompasses decreased mitochondrial biogenesis, increased mitochondrial loss, decreased mitochondrial protein content, and decreased activity of enzymes of the tricarboxylic acid cycle and ETC [20]. Deterioration of mitochondrial function is a sign of metabolic, cardiovascular, renal, neurodegenerative, and inflammatory diseases. Mitochondrial dysfunction has been observed in people with type 2 diabetes (T2DM), obesity, and insulin resistance, accompanied by low oxidative enzyme activity and decreased lipid metabolism in muscle [21-23]. In women with gestational diabetes mellitus (GDM), the number of mitochondria in the placenta was reduced, which was associated with increased mitochondrial fusion that negatively impacted fetal development and offspring health [24]. Diseases closely associated with MetS include T2DM, obesity, and IR [25].

Mitochondrial dysfunction is associated with disruption of cellular homeostasis, which mtDNA mutationstDNA mutations may cause may cause decreased mitochondrial biogenesis/mitochondrial enzyme activity/mitochondrial clearance, disruption of mitochondrial dynamics/bioenergetics, and imbalance of calcium homeostasis. This leads to decreased mitochondrial oxidation, ectopic lipid accumulation, and increased levels of diacylglycerols (DAGs) and ceramides [21], which inhibit insulin signaling. DAGs activate protein kinase Cs (PKCs), which phosphorylate the insulin receptor and inhibit tyrosine kinase activity, thereby inducing IR [26]. Ceramides activate protein phosphatase A2 (PPA2), which dephosphorylates AKT/PKB (protein kinase B) and inhibits AKT-INSR signaling, leading to IR [27, 28]. At the same time, mitochondria can synthesize ceramides due to the activity of ceramide synthase (CerS), which negatively affects mitochondrial respiratory chain functions and mitochondrial fission/fusion activity in T2DM [28]. In addition, disruption of the ETC leads to the formation of superoxide (ROS), which destroys mitochondrial structures and triggers mitophagy [20]. When ROS are overproduced, mitophagy is disrupted, leading to the accumulation of dysfunctional mitochondria, oxidative stress, and apoptosis [29, 30]. High concentrations of ROS produced by mitochondria block the activation of IRS-1 and PI3 kinase in adipocytes, mouse myocytes [31], and 3T3L1 cultures [32]. Disruption of intracellular Ca^2+^ homeostasis is associated with impaired fatty acid metabolism [33], which is associated with alterations in oxidative phosphorylation in the tricarboxylic acid cycle and the development of inflammation [34] in IR and T2DM [25, 35, 36].

Thus, the pathogenesis of the MetS component is directly related to a disturbance of metabolism and mitochondrial functions in various cells and tissues.

#### 2.2.1 ROS Produced by Mitochondria

Characteristic signs of MetS are chronic inflammation and IR [37]. Still, the fundamental component of the pathogenesis of MetS is recognized as systemic oxidative stress [38, 39] - an excessive production of reactive oxygen species (ROS) by mitochondria and nicotinamide adenine dinucleotide phosphate (NADPH) oxidase [11, 40, 41]. These systems are connected directly (mitochondria interact with other organelles; the structural components of NADPH oxidase are incorporated into the mitochondrial membrane) and indirectly (*via* messengers: calcium, cGMP, cAMP, 4-hydroxy-2-nonel, 8-hydroxy-20-deoxyguanosine, microparticles) and form a complex molecular network, the exact processes of which have not yet been researched [41].

ROS exist in the form of free (O_2_^-^, OH, NO, CO_3_^-^, NO_2_, R0/ROO) and non-free radicals (H_2_O_2_, HOCl, ONOO^-^) [42, 43].

#### 2.2.2 Electron Transport Chain (ETC). Mitochondrial Respiratory Complexes

The respiratory chain is located on the inner mitochondrial membrane. It enables the phosphorylation of ADP to ATP using the energy released during the oxidation of electron donors from the tricarboxylic acid cycle (NADH, FADH2) (generation of a proton gradient) [44]. The OXPHOS respiratory chain consists of 5 complexes and two coenzymes. In addition, 13 ETC subunits are encoded by the mtDNA [17].

Complex I is the NADH-ubiquinone oxidoreductase (coenzyme Q oxidoreductase) and consists of 46 subunits (the mtDNA encodes 7 units). The central subunits (there are 14) fulfill a bioenergetic function. Other subunits of complex I are probably involved in electron transport [45]. Complex I oxidize NADH with the quinone coenzyme Q10 (CoQ10), donates 2 electrons to complex III, and pumps 4 protons (H^+^) from the mitochondrial matrix into the intermembrane space.

Complex II has 4 subunits with the succinate coenzyme mechanism of reductase Q and is encoded by nuclear DNA (nDNA). Complex II transfers electrons from succinate to oxidize fumarate to CoQ10 without contributing to the mitochondrial H^+^ gradient [44].

Complex III is a 10-membered CoQ10 cytochrome C oxidoreductase. The central protein of the complex, cytochrome B, is encoded by mtDNA. When electrons are transferred from CoQ10 to cytochrome C, 4 H^+^ is pumped into the intermembrane space [44].

Complex IV acts as a cytochrome C oxidase, transports 2 H^+^ in the opposite direction of the gradient, and reduces O_2_ in the H_2_O molecule. Three subunits of the complex (out of a total of 20) are encoded by the mtDNA. Protons from the intermembrane space pass through the ATP synthase and lead to the synthesis of ATP from ADP and inorganic phosphate [46].

It has been shown that the effective production of ATP by the mitochondria is directly dependent on the ions of the coenzyme Q10 (CoQ10), copper (Cu^2+^), calcium (Ca^2+^), and iron (Fe^2+^). At the same time, oxidative stress is mediated by an imbalance of microelements. The calcium uniporter and the Na^+^/Ca^2+^ exchanger [47] are responsible for the influx of Ca^2+^ into the mitochondrial matrix.

Inefficient oxidative phosphorylation in the mitochondria leads to the formation of ROS when NAD is reduced to NAD^+^ [48, 49]. ROS signaling is involved in normal physiological processes and contributes to defective reactions and metabolic disorders [50, 51]. The superoxide anion produced by mitochondria is converted by superoxide dismutase into H_2_O_2_, which serves as a signaling molecule and powerful oxidant [52, 53]. H_2_O_2_ is necessary for the activation of cellular signaling pathways (angiotensin 2, endothelin-1, aldosterone, prostaglandins) that regulate intracellular calcium homeostasis [50]. ROS activate the transcription factors HIF, PI3K, NF-kB, and MAPK and promote the development of inflammation [54-57].

Obesity, IR, hyperglycemia, chronic inflammation, and dyslipidemia are characterized by an overproduction of ROS [58-62]. Excess food (glucose) leads to increased production of NADH and FADH2 in the mitochondrial electron transport chain [63]. An increased proton gradient leads to the leakage of electrons and the formation of superoxide anions [63]. Excess ROS damages the DNA and the lipids of the cell and organelle membranes [64] and also leads to the formation of toxic lipid peroxides [65]. In obesity, H_2_O_2_ can enter into a Fenton reaction with the formation of hydroxyl radicals - highly reactive ROS [62, 66-68]. It was found that hypertrophied adipocytes release ROS, free fatty acids, and inflammatory mediators. At the same time, the fatty acids activate transcription factors responsible for inflammation in adipocytes and macrophages, increasing the production of ROS and developing low-grade systemic inflammation due to the intracellular accumulation of oxidized components (lipids, proteins, mtDNA, nDNA) damage-associated molecular structures (DAMP) [62, 69].

In MetS, there is a decreased activity of antioxidant enzymes in plasma and increased biomarkers for oxidative damage [70]. Proteins and nucleic acids are oxidized and nitrosylated, and accumulated inactive proteins that overload metabolic pathways lead to DNA damage and activate apoptosis [49, 71]. ROS also alters gene expression in cells, creates an environment of oxidative stress [72], alters insulin signaling, and promote organ damage [73-75].

Obesity, a component of MetS, is associated with excessive fat accumulation or abnormal fat distribution [76, 77] and is a consequence or cause of oxidative stress [78]. Studies (animals, cell cultures) have shown the fundamental role of oxidative stress in the development of obesity through the activation of proliferative function, differential fermentation, enlargement of preadipocytes, white adipose tissue (WAT) and changes in eating habits [59, 78]. In human adipocytes, the activation of pro-inflammatory redox transcription factors (NF-kB, AP-1) and the production of inflammatory mediators that increase the production of ROS are observed in connection with obesity [79, 80]. Oxidative stress (ROS) and inflammatory factors disrupt the insulin signaling pathway in the cell, cause IR [62], and are observed in diseases associated with obesity: atherosclerosis and T2DM [81, 82].

Malondialdehyde (MDA) is formed by the degradation of polyunsaturated fats by ROS and is a marker of oxidative stress and peroxidation. A significant increase in MDA in plasma was observed in patients with MetS [83]. In addition, high concentrations of oxidation markers (8-OHdG, 4HNE, protein carbonyl), including MDA [84, 85], were detected in the placenta of obese animals (mice, pigs). In addition, placenta from obese women showed low mtDNA content, decreased electron transport chain complex I activity, and higher than normal levels of mitochondrial H_2_O_2_ [61]. The placenta of women with T2DM was characterized by reduced activity of complex II and III compared to controls [85].

Thus, disruption of the mitochondrial respiratory chain leads to overproduction of ROS, mitochondrial dysregulation, and reprogramming of cellular metabolism, which mediates the development of MetS.

## 3 EPIGENETIC REGULATION OF MITOCHONDRIAL ACTIVITY: FOCUS ON MICRORNAS

It is known that normal mitochondrial function is achieved through the control of mitochondrial protein synthesis, which is regulated by a variety of epigenetic mechanisms, of which miRs are the most important [86, 87].

### 3.1 Micrornas

miRs are small non-coding RNA molecules (oligonucleotides, 18-25 nucleotides) that act as post-transcriptional regulators of gene expression by targeting mRNAs.

The biosynthesis of miRs begins with the transcription of nuclear DNA into a primary transcript (pri-miRs) [87]. Nuclear RNA polymerase II produces primary miRs (pri-miRs), which are processed into precursor miRs (pre-miRs) in the nucleus by the DROSHA protein and DGCR8/PASHA. a hairpin-like structure characterizes pre-miR and is more stable compared to primiR. Pre-miR is exported into the cytoplasm by exportin 5, which recognizes a 2-nucleotide hairpin loop overhang [88]. The pre-miR is then cleaved (spliced) by the RNase (nuclease) Dicer, resulting in a double miR structure with incomplete base pairing [18]. Normally, one strand (strand) is selected for its greater thermodynamic stability (the other strand is destroyed) and binds to the Argonaute 2 (AGO2) protein, resulting in a mature miR of 20-22 bp in length [17]. There are cases where both miR chains are retained and subsequently exert biological functions [17].

AGO2 promotes the incorporation of bound miR into the RNA-induced splicing complex (RISC) [18]. In addition, miRs associated with AGO2 are distributed to other cellular compartments, such as the nucleus or mitochondria, or excreted from the cells as part of exosomes [17].

Once miRs are integrated into the RISC complex, they can bind to mRNAs by in-complete base pairing (3´ untranslated region (UTR)), leading to degradation of the mRNA or inhibition of its translation [17]. Because target gene mRNA recognition does not require fully complementary base pairing, one miR can modulate multiple mRNAs, and one miR can be the target of numerous miRs [17].

A dynamic (changing in response to cellular stress) presence of miRs in different cellular organelles (nucleus, endoplasmic reticulum (ER), mitochondria) has been discovered. Mitochondria-associated ER membranes (MAMs) are organelle contact sites and contain a large number of miRs [89]. Furthermore, miRs localized in mitochondria are involved in the functional regulation of the organelle [90-93].

### 3.2 What are Mitomirs

The last decade has been a watershed in terms of the discovery of miRs in mitochondria, now referred to as mitomiRs [94]. An important role of mitomiRs in local mitochondrial protein synthesis and regulation of mitochondrial function has been demonstrated [94]. A study by Kren *et al.* (2009) identified mitomiRs in rat liver (miR-130a, -130b, -140, -320, -494) that are involved in the expression of genes that regulate apoptosis, proliferation, and differentiation of hepatocytes [95]. In addition, Barrey *et al.* (2011) confirmed the presence of miR-365, pre-miR-302a, pre-let-7b in human mitochondria isolated from myocytes [96].

Pre-miRs and mature miRs were found in mitochondria, suggesting the possibility of intramitochondrial synthesis of miRs. It is possible that some pre-miR sequences are processed in the mitochondria and can synthesize mature miRs that exert direct activity on mitochondrial transcripts or are transported into the cytosol to interfere with genomic mRNA [97].

It is hypothesized that there are multiple ATP-dependent pathways for the import of miRs into the mitochondria, which are encoded by the nucleus and differ depending on the type of miR. Nuclear genome mapping has revealed a number of tissue-specific miRs that are directly related to mitochondrial functions or mitochondrial pathologies [97].

It has been found that miRs originating from the nucleus are exported to the cytosol and imported into the mitochondria, where they interact with some mRNA molecules originating from the mitochondrial genome. At the same time, the mitochondrial genome (mtDNA) can produce some miR molecules that affect mitochondrial transcripts [97]. MitomiRs were found to have thermodynamic properties and sizes distinct from canonical miRs and to be expressed almost exclusively by nuclear genes at loci related to mitochondrial function. The role of miRs in the initiation of mitochondrial translation is well known: increased protein synthesis and ATP production.

A special feature of miRs is the ambiguity of their effect on the expression of target genes. For example, miR-1, which enters the mitochondria of myocytes, stimulated rather than suppresses the translation of transcripts encoded by the mitochondrial genome. This suggests a positive regulatory function of miRs in mitochondria and the presence of novel, previously unexplored connections between the nuclear and mitochondrial genomes that are regulated by miRs [97].

### 3.3 Mechanisms of Mir Transport into the Mitochondria

Mitochondria-associated miRs (mitomiRs) can be divided into three types: (1) mi-tomiRs that originate from the nuclear genome, mature in the cytoplasm and target cellular genes that modulate mitochondrial functions (nuc-MiRs); (2) mitomiRs that are translocated to the mitochondria to regulate mitochondrial genes and functions (nuc-mitomiRs); (3) mitomiRs that are transcribed from the mitochondrial genome and target mitochondrial genes (mt-mitomiRs) [98].

Mammals possess four Argonaute proteins (Ago1-4) [99] that are involved in the processing, stability, and function of miRs [100].

#### 3.3.1 Argonaute 2

Argonaute 2 (AGO2) is an important mitochondrial protein involved in miR bio-genesis [101, 102]. AGO2 is thought to have a mitochondrial sequence that targets its amino acid region and contributes to its localization in mitochondria [18]. AGO2 levels and disruption of AGO2 function (phosphorylation at specific serine residues) affect the translocation of miRNA to mitochondria [18]. AGO2 proteins interact with mature miR to form a ribonucleoprotein complex, the RNA-induced silencing complex (RISC), which stabilizes the binding of miR base pairs to target mRNA and promotes transcriptional repression and mRNA degradation [103]. RISC includes other RNA-binding proteins: GW182 and TRBP1/2. The presence of the DICER protein in the RISC complex is controversial [93].

The transport of RISC into the mitochondria occurs through the coordinated actions of the sorting and assembly machinery (SAM50), the translocase outer mitochondrial membrane 20 (TOM20), and the translocase inner mitochondrial membrane (TIM) [104]. MiR transfer into the mitochondria depends on the expression level of the AGO proteins and their phosphorylation status [105]. Phosphorylation of AGO2 at Ser387, Ser824, and Ser834 can interfere with the binding of mRNA to miRs in RISC [106, 107]. The interaction between mitochondria and AGO2-miR-containing processing bodies (P-bodies) is shown [108]. Phosphorylation of AGO2 at Ser387 can initiate AGO2-miR entry into the P-body through dissociation from the RISC complex and activation of the KRAS-MEK [109] and ANKRD52-PPP6C [106] signaling pathways. At the same time, P-bodies contact mitochondria and the ER and regulate the decay/storage of mRNA and the import of miR [18, 108]. P-bodies can promote the import of miRs into mitochondria [18]. In addition, AGO2 is involved in the encapsulation of miRs in exosomes [110, 111].

#### 3.3.2 Protein GW182

The protein GW182 has been found to play a crucial role in the maintenance of the AGO2-miR complex by binding to its partner in RISC [112]. GW182 has two specific tryptophan residues that bind directly to the PIWI domain of the AGO protein to form RISC [113]. Cellular stress and decreased Akt phosphorylation lead to the displacement of GW182 from RISC in the cytoplasm [114]. It is hypothesized (further research is needed) that there is a unique biological mechanism that allows the AGO2-miR complex to enter the mitochondria against a background of dissociation of GW182 from RISC in the cytoplasm. AGO thus acts as a RISC complex in the cytoplasm [115].

Liver-specific Ago2 deficiency in mice increases mitochondrial oxidation and exacerbates obesity-related pathophysiology. Hepatic Ago2 regulates PPARa functions and oxidative metabolism. Hepatic Ago2 function has been found to be closely linked to PPARa, providing a link between Ago2-mediated RNA silencing and mitochondrial functions for oxidation and obesity-related pathophysiology [116]. Hepatic RNA silencing mediated by Ago2 regulates energy production and energy expenditure and influences energy metabolism in the pathogenesis of obesity. Liver-specific deletion of Ago2 has been found to increase mitochondrial oxidation and ATP consumption associated with mRNA translation, leading to activation of the AMPK signaling pathway and promoting obesity-related pathophysiology [117]. Studies in mice have shown that Ago2 expression is associated with obesity and increased adipose tissue. Increased levels of Ago2 inhibited AMPKa expression in association with high expression of miR-148a [118].

#### 3.3.3 PNPase

In addition to the endonuclease AGO2 and the RISC complex, the transfer of miR into mitochondria is controlled by the protein enzyme 3´-5´-exoribonuclease and the polypolymerase polynucleotide phosphorylase (PNPase), which is located on the inner (and outer) membrane of mitochondria and projects into the intermembrane space [18, 105, 119]. PNPase helps the RNA to maintain the correct structure to migrate through the TIM/TOM pore and then returns the RNA to its previous conformation when the RNA reaches the mitochondrial matrix. In addition, PNPase levels influence the import of MitomiR [120]. It is assumed that PNPase can transport miRs with a certain number of nucleotides at the 3'-end (up to 2 nucleotides) into the mitochondria [121]. Disruption of PNPase (pntpl) gene expression resulted in mitochondrial dysfunction and disruption of organelle morphologic structure, which impaired the import of miRs that control the transcription/translation of mitochondrial electron transport chain (ETC) proteins in hepatocytes [120, 122]. The results of co-immunoprecipitation showed a relationship between PNPase and AGO2 [120]. At the same time, PNPase activity was detected in the recognition and transfer of pre-miRs with a specific configuration of the secondary “stem-loop” structure [121].

#### 3.3.4 VDAC

The voltage-dependent anion channel (VDAC) is another potential miR import channel. VDAC is located at the outer mitochondrial membrane and facilitates the transfer of small non-coding RNAs into the mitochondria. The ability of VDAC to bind to tRNA in plant cells has been demonstrated [123]. However, the exact role of miRs in trafficking remains to be investigated.

Within the mitochondrial matrix, microRNAs can bind to mRNAs produced in the mitochondria and influence the expression and regulation of mitochondrial genes. There is usually a decrease in the expression of target mRNAs as a result of mRNA degradation or decreased ribosome occupancy due to decapping induced by deadenylation and altered cap protein binding. This prevents the mRNA from reaching the translation enzymes. However, in some cases, RISC binding increases gene expression by competitively repressing stronger RNA repressor proteins [18].

The above methods of transporting miRs from the cytoplasm to the mitochondria, therefore, require further detailed investigation in terms of their potential utility for the development of therapeutic constructs targeting mitochondria and carrying specific miRs.

## 4 THE EFFECT OF MIRS (MITOMIR) ON MITOCHONDRIA IN METABOLIC SYNDROME

MetS and obesity are pathological conditions directly related to mitochondrial dysfunction and associated with the development of oxidative stress and systemic inflammation [123, 124]. Excessive intake of fatty acids leads to disruption of cellular homeostasis, production of ROS, alterations in intracellular signaling (inhibition of tricarboxylic acid cycle aconitase), cell apoptosis in adipose and muscle tissue, and promotes fat deposition and the development of IR [11]. A number of reviews have been published describing the important role of miRs in the pathogenesis of MetS components [125-134], although the role of potential mitomiRs in these pathologies is poorly characterized, with a focus on their targeting mitochondria.

We believe that comparing the role of miRs that enter mitochondria and regulate key target genes that modulate organelle functions and are involved in the development of MetS/obesity with miRs that are directly related to the functional potential of mitochondria in metabolic disorders will provide us with a more comprehensive view of the problem of MetS and identify potential targeted therapeutic targets and important candidates for future research.

### 4.1 Electron Transport Chain and Mitomirs

It is known that the function of the electron transport chain depends on the assembly of the mitochondrial iron-sulfur (FeS) cluster through the activation of the enzyme ISCU 1/2. ISCU is a key element of the ancient mechanism that catalyzes the assembly of FeS, which is critical for the function of aconitase (a member of the tricarboxylic acid cycle) and mitochondrial ETC complexes I, II, and III [135].

It was found that the targets of miR-210 (as well as miR-128) are the mRNA of the genes of the mitochondrial ETC components: NADH dehydrogenase (ubiquinone) 1α-subcomplex 4 (NDUFA4), succinate dehydrogenase complex, subunit D (SDHD), cytochrome c oxidase assembly homologue 10 (COX10) [135, 136].

The regulation of FeS miR-210 [137] occurs by inhibiting the translation of the cytoplasmic isophore ISCU2 and reducing the activity of ECT. MiR-210-5p shows the highest activity under conditions of hypoxia [138], ROS [139], and iron deficiency, and the miR-210-5p gene is a target of HIF-1x [140]. MiR-210 suppresses ISCU and promotes mitochondrial dysfunction in rat neurons [141] and is involved in mitochondrial potassium channel opening *via* the HIF-1α/miR-210/ISCU mechanism [142]. Studies in cell lines (A2058, G361, 293T) showed activation of the miR-210/ISCU/ROS pathway [140]. miR-210-5p is mitomiR, as evidenced by its localization in the mitochondria of human myocytes [17].

In addition, miR-210 targets complex II subunit D of the succinate dehydrogenase (SDH) complex and complexes III, IV, and V and interferes with COX10 mRNA [143-145].

High expression of miR-210 is associated with the development of obesity/(neuro/meta)inflammation/IR [139, 146-150] and has been observed in resident macro-phages of adipose tissue [146], peripheral blood [151] and placenta [152].

The ETC complex IV has 3 subunits that are encoded by the mtDNA (mt-COX1, mt-COX2, mt-COX3). The remaining subunits are encoded by nuclear DNA and translocated into the mitochondria.

MiR-338-5p targets the expression of the nuclear-encoded subunit IV of cytochrome c oxidase (COX IV), which regulates the assembly of complex IV and its respiratory potential [153, 154]. Also, it affects the subunits of the ATP synthase complex [17, 153]. MiR-338-3p is expressed in MSCs and determines the adipogenic direction of cell differentiation [155]. Reduced expression of miR-338-3p mediates TNF-dependent development of liver IR in mice receiving HFD [156].

It was found that miR-181c [157-159] and miR-1 [160] can interact with mt-COX1. Both miRs were found in mitochondria and regulated organelle dynamics and cell differentiation/proliferation [16, 47, 96, 157, 159, 161-164].

Obesity and MetS are associated with high expression of miR-181c [165, 166], which can lead to the development of heart failure (in mouse cardiomyocytes) *via* the regulation of target genes (Sp1, MICU1) [158, 165].

Also, miR-127-5p, miR-101-3p, and miR-378 [167] are involved in the regulation of ATP synthase subunits. In addition, the mitomiR class includes: miR-378-3p [163], and miR-101-3p (hepatocytes) [17].

In obesity, miR-378 was associated with IR [168] and inhibited p110a (a regulator of glucose and fatty acid metabolism) by antagonizing the coactivator gamma receptor 1-β. The adipogenic [169] and protective role of miR-378 against obesity [170, 171] and MetS [172] has been demonstrated through modulation of carbohydrate and lipid metabolism [173] against a background of induction of leptin/proinflammatory mediators in human adipocytes (when miR-378 is expressed) [172, 174].

RNA sequencing analyzes in MSCs from animal (porcine) adipose tissue revealed a number of miRs whose high expression was associated with dysregulation of mitochondrial functions (structure) and COX IV function in obesity [175]. The expression of the investigated miRs (miR-196a, miR-27b, miR-504, miR-212-5p, miR-let-7c, miR-210-3p) was also increased in MSCs from adipose tissue of obese patients [175, 176].

Another mitomiR that regulates the activity of the respiratory complex and is involved in the development of obesity and MetS is miR-762. Nuclear miR-762 translocates into the mitochondria [115]. It reduces the protein concentration of endogenous NADH dehydrogenase subunit 2 (ND2) (in cardiomyocytes), an important subunit of the mitochondrial respiratory chain that is required for ATP production [177]. In addition, miR-762 targets the AGAP-2 gene, a negative regulator of UNC5B and netrin-1 signaling [178, 179], which plays a protective role against obesity (and the development of inflammation) by inhibiting the migration of macrophages into adipose tissue [178] and regulating innate immune system genes (RNase7, ST2, Rab5a) [180].

The genes of the mitochondrial respiratory chain are also regulated by mitomiR Let-7b [181]. Let-7b-5p promotes HFD-induced obesity (suppresses white-to-beige adipocyte transition) and hepatic steatosis by reducing mitochondrial oxidative phosphorylation [182]. In addition, obesity during pregnancy is associated with high expression of miR let-7d in the peripheral blood and placenta [183].

Thus, there are a variety of miRs that are localized in the mitochondria and can regulate their respiratory functions through targeted interaction with specific regions of mtDNA. Considering the important role of mitochondrial dysfunction with emphasis on the overproduction of ROS in MetS (and its components), the identification of new mitomiRs will allow us to identify their new functions/roles in the pathogenesis of these diseases and apply the knowledge gained in the diagnosis and treatment of MetS.

### 4.2 Tricarboxylic Acid Cycle

Glycolysis produces pyruvate, which is converted by pyruvate dehydrogenase (PDH) into acetyl-CoA, which enters the tricarboxylic acid cycle in the mitochondrial matrix. As a result, redox equivalents are formed that enter the ECT and trigger an electrochemical proton gradient.

The activity of the PDH enzyme depends on a non- catalytic component, subunit X, which is inhibited by miR-26a and controls glucose metabolism in cells [184]. An important protective role of miR-26a against obesity was found by blocking adipogenesis (Fbxl19 gene) [185] and differentiation of preadipocytes into human beige adipocytes (ADAM17 gene) [186]. Obesity promotes decreased expression of miR-26a in the liver and, indirectly, the development of IR, high glucose production, and fatty acid synthesis (humans, mice) [187]. In addition, miR-26a/b-5p modulates the expression of cytochrome c oxidase subunit 5a, which is reduced under hypoxic conditions (cardiomyocytes) [188]. This could indicate a possible localization of miR-26 in mitochondria.

Pyruvate dehydrogenase kinase 1 (PDK1) is considered a key enzyme in the transition from glycolysis to the tricarboxylic acid cycle by inactivating PDH and converting oxidative phosphorylation to the Warmburg effect, leading to increased lactate production [189]. Inhibition of PDK1 expression resulted in a decrease in lactate, ATP, ROS accumulation, mitochondrial damage and increased apoptosis.

It was found that the target of miR-128-3p is PDK1 (in cancer cells) [189]. At the same time, against the background of MetS in hepatocytes (rats, mice), a decrease in miR-128-3p and disruption of insulin signaling transduction (IRS-1), biogenesis of fatty acids (FOXO1), triglycerides and lipoproteins [190] were observed. However, in another study on mouse liver cells, increased expression of miR-128-3p was found to target the inhibition of PPARy [191]. Further studies will determine the detailed role of miR-128 in mitochondrial physiology in MetS and obesity.

The first step of TCA is catalyzed by citrate synthase (CS), which couples acetyl-CoA to oxalic acid.

MiR-494-3p regulates the expression of this enzyme. In addition, miR-494-3p has a modulatory effect on mitochondrial biogenesis by activating the transcription factor PPARy *via* its coactivator PGC1-α and controlling downstream targets (TFAM, Ucp1) in 3T3-L1 cell cultures, mature adipocytes, and hepatocytes (mice) [192-194]. In addition, a study in mouse hepatocytes demonstrated the presence of miR-494-3p in mitochondria, confirming the important regulatory role of this miR not only at the nuclear level but also as mitomiR [17].

Regulators of the CS enzyme are also miRs (miR-152-3p, miR-148a-3p, miR-148b-3p, miR-299- 5p, miR-19a-3p, miR-19b-3p, miR-122-5p, miR-421), some of which have been found in mitochondria [17].

The FeS-dependent enzyme aconitase mediates the isomerization of citrate to form isocitrate. As mentioned above, mitomiR-210-5p also negatively affects iso-citrate production by decreasing the activity of the enzyme ISCU2 [17].

In addition, miR-210-5p interferes with the formation of fumarate from succinyl-CoA by decreasing the activity of succinyl-CoA dehydrogenase (SDH) [135]. Overall, miR-210-5p leads to a loss of mitochondrial membrane potential and mitochondrial dysfunction [17]. The role of miR-210 in the development of metabolic diseases, including MetS components, has been repeatedly demonstrated [139, 146-150].

The next enzyme in the tricarboxylic acid cycle is succinate-Co-A-GDP ligase, which forms the β-subunit (SUCLG2) and catalyzes the conversion of succinate to succinyl-Co-A.

It was found that miR-124 represses the expression of SUCLG2 [93]. In db/db mice, a decrease in the expression of miR-124-3p and induction of the target gene FOXQ1 and the development of mitochondrial dysfunction in the epithelial cells of the renal tubules were observed [195]. Against the background of obesity and T2DM, a reduced expression of miR-124-3p was observed in the peripheral blood of patients (even after bariatric surgery/gastric bypass) [196].

The TCA, which takes place in the mitochondrial matrix, is therefore, directly related to mitomiR. However, the exact effects of mitomiR on specific parts of the Krebs cycle in different cell types in MetS/obesity remain to be investigated.

### 4.3 Mitochondrial Fission and Fusion

#### 4.3.1 Mitochondrial Fission

The mitochondrial equilibrium depends on two differently oriented processes: Fusion and fission. The central mediator of mitochondrial fission is the cytosolic dynamin GTPase-related protein I (DRP1). The translocation of DRP1 to the outer mitochondrial membrane is regulated by the adapter protein FIS1, the mitochondrial fission factor (MFF), and the mitochondrial dynamin proteins (MID49, MID51) [197], which leads to the formation of a constricting ring structure around the mitochondria and the division of the organelle into two parts.

The mitochondrial protein DRP1 is a target for miRs: miR-34a, miR-21-5p, miR-181a/b, miR-30a-5p, miR-499-5p, miR-199b-5p, miR-203a-3p; the adapter protein MFF is a target of miR-761 and miR-27, and the FIS1 protein is regulated by miR-484 [17, 198-202].

An important function in the development of obesity and the regulation of DRP1 is performed by mitomiR-34a [163, 203-206]. MiR-34a has many target genes, including endogenous hormone core, FGF21 (β-Kloto) [207], SIRT1 [208-210], PINK1 [211], cytochrome C [212, 213], transfer protein kinase C (PKC) signaling [214], that regulate energy metabolism and are involved in the development of metabolic diseases: Obesity, IR, NAFLD [206, 215-219]. High expression of miR-34a is associated with impaired ETC function (due to targeting of NDUFS8, COX5b, ATP5a1) [220] and induction of the inflammatory transcription factors NF-kB, STAT1, CREB [221-224].

Thus, miR-34a in mitochondria is involved in replicative and inflammatory cell aging [225], which is associated with obesity and metabolic disorders.

Another mitomiR that targets DRP1 and is associated with the development of MetS and obesity is miR-21 [226]. Obesity associated with lipotoxicity and IR [227] is characterized by high expression of mitochondrial miR-21 in adipose tissue, liver and peripheral blood (in humans and rats) [163, 228-231], leading to activation of VEGF-A signal-ing, p53, TGF-β1 [228], NF-kB, c-Jun and AKT [232, 233].

MitomiR-181a [163] targets DRP1, BDNF [234, 235], cytokine expression (TGFβ, IL-10, IL-6, IL-1, TNFa), and NF-kB signaling pathways (TLR/NF-kB) [236] and is directly associated with the development of inflammatory/oxidative stress [237-240] in obesity [241] and MetS [241]. The direct presence of miR-181a in mitochondria is associated with the regulation of the biochemical/morphological processes of replicative cell senescence (observed in obesity, MetS), which is associated with suppression of Bcl-2, induction of permeability transition pore opening, activation of caspases (1/3), regulation of apoptosis and conversion of LC3-I to LC3-II [242].

MitomiR-181b also inhibits the activity of DRP1, a phosphatase that phosphorylates Akt at Ser473 [243], BDNF [244, 245], and PTEN [158]. In obesity, an increase in the level of miR-181b in blood plasma was observed [205], but a decrease in the endothelial cells of adipose tissue (mouse) [243].

DRP1 is a target gene for miR-30a [246, 247]. MiR-30a targets the expression of p53 and DRP1 target genes (subcytes) and thus prevents glucose-induced mitochondrial fission [198]. Target molecules for miR-30a are PPARy, ADIPOQ, FABP4 (adipose tissue) [248], STAT1 [249]. The expression of miR-30a in adipose tissue is reduced in the context of obesity and diabetes [249, 250].

The role of miR-499-5p in the regulation of DRP1 has been demonstrated [251, 252]. The target genes for miR-499-5p are PTEN [253], which regulates insulin signaling and glycogen synthesis in the liver (db/db mice); PRDM16, UCP1, PGC1α (a marker of adipogenic differentiation) [254]; Fnip1, AMPK-interacting protein [255]. Obesity and HFD lead to increased hepatic levels of miR-499-5p [253].

MiR-199b-5p-dependent regulation of AKAP1 (A-kinase anchoring protein 1)/DRP1 was detected [200]. In human umbilical vein cells (HUVEC) treated with oxidized LDL (ox-LDL), there was a decrease in miR-199b-5p expression and an increase in ROS [200].

The results of bioinformatic analysis predicted the target for miR-203a-3p - DRP1 [202, 256].

It was found that MFF is a target for miR-761 [257] and the expression of miR-761 increases in myocytes in the background of IR [258].

In addition, MFF mRNA is a direct target of miR-27 [259]. MiR-27 is involved in adipogenesis [260] and reduces the expression of Prohibitin (PHB) [261, 262], and PPARy [263, 264].

Also, miR-200a-3p [265] has an effect on MFF. Overexpression of miR-200a is associated with impaired leptin/insulin signaling (mouse hypothalamus) [266], appetite regulation in obese children [267], and the development of NAFLD [268].

MiR-484 suppresses the translation of the FIS1 protein [269, 270]. Other targets of miR-484 are SMAD7, YAP1, BCL2L13 [270], Sorbs2 [271]. High expression of miR-484 has been reported to be associated with impaired glucose transport, IR, and metabolic dysfunction [272].

PGC-1a and TFAM are known to play an important regulatory role in mitochondrial fission and biogenesis [17]. Thus, miR-128 reduced mitochondrial biogenesis in human myoblasts (C_2_C1_2_) through suppression of PGC-1a, TFAM, nuclear respiratory factors (NRF1/2) and upregulation of DRP1 [136]. Inhibition of miR-128 was associated with an increase in mitochondrial mass and ATP production (reversing the effects of metabolic perturbations) [136]. At the same time, it was observed that high expression of miR-128 in the background of overeating (in mice) leads to metabolic mismatch and fat deposition [273]. PGC-1a also acts as a target for miR-23 [274] and miR-696 [275, 276], which play an important role in the pathogenesis of obesity [277, 278].

Thus, there are a number of miRs that influence mitochondrial biogenesis by regulating the activity of mitochondrial fission proteins. Further studies will make it pos-sible to identify among them miRs that can translocate into the mitochondrial matrix and have additional functions. This will provide the opportunity to understand the complex relationships and signaling pathways involved in MetS and to identify the most effective miRs for the development of targeted drugs against metabolic diseases.

#### 4.3.2 Mitochondrial Fusion

The fusion of the membranes of two mitochondria is called thermonuclear fusion and has a protective function that helps to maintain healthy mitochondria and prevent apoptosis. Mitochondrial fusion begins with the contact of the OMM proteins: Mitofusin 1, 2 (MFN1/2).

MiR-106b, miR-140, miR-17-5p, and miR-15a-5p reduce the expression of MFN1/2 and are associated with mitochondrial dysfunction [279, 280]. An important role for MFN1/2 in mitochondrial/energy metabolism and IR has been found in obesity and diabetes [281]. Obesity and T2DM have been associated with low MFN2 expression and suppressed oxidative phosphorylation in skeletal muscle [282, 283]. Deletion of MFN2 in the liver and muscle (in mice) led to mitochondrial fragmentation, IR, and increased gluconeogenesis in the liver [284].

In mitochondria, miR-92a-3p binds to Mfn1, regulates cell viability (H9c2) and repairs damaged mitochondria [285]. MiR-92a-5p positively modulates the expression level of the mitochondrial cytochrome b gene (mt-Cytb) in cardiomyocytes of db/db mice, and reduces ROS production and lipid deposition [286]. The targets of miR-92a are members of the SMAD family [287], KLF2, which are involved in cell proliferation, differentiation, apoptosis, and inflammation [288, 289]. MiR-92a also controls the differentiation of adipocytes [287, 290, 291]. In obesity, high expression of miR-92 was observed in blood serum [292, 293] and in blood leukocytes [294].

OPA1 (optic atrophy protein 1) is known to mediate fusion of the inner mitochondrial membrane. The long-form L-OPA1 is cleaved by mitochondrial proteases (YME1L1, Oma1) into the short form S-OPA1. L-OPA1 is located in the inner mitochondrial membrane, while S-OPA1 is localized in the mitochondrial matrix. L-OPA1 thus targets the fusion of the inner mitochondrial membranes, while S-OPA1 promotes mitochondrial fission [295].

OPA1 is involved in the development of obesity by regulating lipolysis [296, 297]. The translocation of OPA1 from mitochondria to lipid droplets was observed during adipocyte differentiation [298]. Furthermore, the absence of Oma1 led to the development of obesity in mice [299].

Suppression of OPA1 is associated with the activity of miR-16 [300], miR-664a-5p [301], and miR-361-5p [302]. High expression of these miRs has been found to be involved in the development of obesity [303-305] and replicative aging [306].

Thus, the epigenetic component based on the action of miRs plays an important role in regulating the process of mitochondrial fusion. A detailed investigation of the targets for already known mitochondria and the identification of miRs localized in mitochondria will provide further opportunities for the development of a molecular therapy with low toxicity in the fight against metabolic disorders.

## 5 THE MEDICAL POTENTIAL OF MITOMIRS

Drugs for the treatment of diseases associated with mitochondrial dysfunction (T2DM) and neurodegenerative pathologies can modulate the activity of the ETC complex (electron transport chain) by increasing the availability of substrates or inducing mitochondrial biogenesis [307]. Several RNA-based approaches have been investigated at the cellular level to target mitochondrially encoded transcripts specifically. RNA therapeutics reduce the risk of genomic integration and can be pharmacologically controlled due to their transient mechanisms of action [308], which is an advantage over DNA therapies. In particular, RNA-based therapeutics can directly target a large part of disease pathogenesis (*e.g.*, by replacing missing proteins due to mtDNA deletions).

Current classes of RNA therapeutics include antisense oligonucleotides (ASOs), small interfering RNA (siRNA) and mRNA therapeutics [309] that target the inhibition of transcription of specific mitochondrial mRNAs [310].

ASOs are single-stranded oligonucleotides that bind complementarily to the target mRNA/damaged mRNA and cause degradation, altered splicing, or inhibition of translation [311]. siRNAs are double-stranded oligonucleotides that inhibit gene expression by RNA interference (silencing of gene expression), which is mediated by RISC in the cytoplasm [312, 313]. There are four approved siRNA-based drugs (Patisiran, Givosiran, Lumasiran, and Inclisiran).

The development of large coding mRNAs (more than 4000 nt) with therapeutic potential has become an urgent area following the successful use of mRNA vaccines *in vivo* [314-316]. The introduction of mRNA is a promising method for genome editing systems such as CRISPR-Cas9 (clustered regulatory interspaced short palindromic re-peats) and small guide RNA (sgRNA) [317, 318].

The introduction of miRs, mitomiRs, and ASOs into mitochondria to increase/decrease mitochondrial gene expression to achieve targeted outcomes is an attractive area of research [319].

There are two strategies for introducing mRNA into mitochondria: direct and indirect delivery.

### 5.1 Direct Delivery

Direct delivery of mRNA to the mitochondria, where a carrier targeted to the mitochondria releases the RNA directly into the mitochondrial matrix.

Direct transport of mRNA into the mitochondria has been made possible by the development of cell-penetrating RNA nanocarrier systems that target the mitochondria. These carriers include liposomal, inorganic, polymeric, or DQAsomes [320-326].

The development of drug carriers for mitochondria is based on a characteristic feature of mitochondria-a negative charge created by the potential difference between the inner and outer mitochondrial membranes, which ensures the movement of protons for the production of ATP [321, 327]. Targeting to mitochondria is often achieved with the help of delocalized lipophilic cations (DLCs): triphenylphosphonium cations (TPP+), mitochondria-penetrating peptides, mitochondria- targeting signal peptides, RNA aptamers and DQAsomes [328-330].

TPP+ is a lipophilic cation that accumulates on the inner mitochondrial membrane [331]. Some drugs (AP39, Mito-Vit-E, Mito-Q, SkQ1, Mito-TEMPOL) are transported into the mitochondria *via* TPP+ [321].

A polymer-based nanocarrier system consisting of a 5th generation poly(amidoamine) dendrimer (PAMAM(G5)-TPP) conjugated to TTP and carrying Let-7b colocalizes with mitochondria in a non-small cell cancer cell line lung A542 and decreases the expression of the mt-mRNA COX1/2 [181]. However, the expression of cytoplasmic mRNAs KRAS and p21 was impaired, indicating a partial release of let-7b in the cytosol [181]. At the same time, high concentrations (10 µM) of TPP+ can have a negative/damaging effect on mitochondria by inducing proton leak and depolarization of the mitochondrial membrane [320, 332].

An alternative strategy to deliver substances (nanostructures) into the mitochondria is to utilize the specific physicochemical properties of the drugs. The structural similarity between mitochondria and bacteria formed the basis for the development of hemigramicidin (GS) emitters that are conjugated with a ROS scavenger and reduce oxidative damage to cells [320].

Considering the presence of the protein cardiolipin in the mitochondrial membrane, a non-toxic (1 mM) SS peptide (Seto-Schiller) was developed that can penetrate the plasma and mitochondrial membranes and effectively remove ROS by inhibiting lipid oxidation. The second phase of clinical trials with SS-31 [333] confirms the potential immediate use of this peptide as an effective drug [320, 321, 334].

A non-cationic liposome-based nanocarrier, MITO-porter, has been developed that enters cells by pinocytosis mediated by high-density octa-arginine on the surface of the nanocarrier [335]. In the cell, the MITO-porter leaves the endosome and binds to the mitochondria, fuses with the mitochondrial membrane, and delivers its cargo specifically into the mitochondrial matrix [336]. The successful transport of CoQ10 into the mitochondria of the liver of mice was mediated by the CoQ10-MITO-porter system [337].

DQAsomes are a unique drug delivery system due to their dequalinium content, which has the property of self-assembling into liposome-like cationic vesicles that target mitochondria [338, 339].

Polymer nanoparticles of poly(lactic, glycolic acid) (PLGA) are biodegradable and biocompatible [340]. They are being actively investigated in mixed platforms of targeted nanocarriers targeting mitochondria in obesity and cancer (PLGA-b-PEG (polyethylene glycol)-TPP NPs) [341].

### 5.2 Indirect Delivery

Indirect delivery in which RNA molecules are modified by endogenous mitochondrial RNA import determinants and enter the cytosol of the target cell with the aid of a conjugate/carrier. The advantage of this strategy is the successful use of nanocarriers and conjugate systems for cytosolic delivery of RNA into the cell (LNP, GalNAc) in drugs, including vaccines, miR, and ASO [342, 343] in humans.

A limitation of this approach is that these constructs require additional modifications to transfer their content into the mitochondrial matrix specifically. However, this problem can be partially solved by the methods that have been developed to modify nanocarriers/conjugates based on the molecular mechanisms of RNA import into human mitochondria (5S p RNA, RMRP (RNA component of RNase MRP), RPPH1 (RNA component of RNase, RNA H1), SAMMSON (survival-associated oncogenic non-coding RNA specific for mitochondrial melanoma), hTERC, GAS5 (growth arrest specific 5), selected tRNAs and microRNAs [344-347]. Given the lack of understanding of the process of RNA import into the mitochondria and the likely tissue/cell specificity, the development of new molecular constructs with therapeutic potential is currently limited. In addition, a number of questions remain unresolved regarding the delivery of an RNA therapeutic to the target tissue and the subsequent delivery of an RNA therapeutic into the mitochondrial matrix of the cell [311].

## CONCLUSION

Mitochondria are the most important organelles for the life of a cell. They primarily regulate energy homeostasis under normal and pathological conditions. Considering that mitochondria differ in the characteristics of biogenesis (fusion/fission) and that the tricarboxylic acid cycle occurs in them and the production of ATP and ROS takes place, the disruption of these processes in response to changes in cellular energy costs plays an important role in the pathogenesis of metabolic disorders. The maintenance of the functional potential of mitochondria is directly related to epigenetic factors, miRs and mitomiRs. It is assumed that an important role in the development of MetS/obesity is played by microRNAs, which have a pro-/anti-inflammatory potential and can penetrate the mitochondrial matrix. They regulate the activation of genes responsible for the structural integrity and functional potential of mitochondria in various organs, including the brain [2]. The mitochondrial dysfunction associated with the development of MetS and obesity, not only in peripheral organs but also in the central nervous system, represents a promising therapeutic target. Given the important role of miRs in the development of these pathologies, studying their biology and targets directly in the mitochondria is an urgent goal. The methods that have been developed to deliver agents into the mitochondria, including nanostructures and combined structures [320], have not been sufficiently explored at this stage of scientific development, as there is little data on the toxic effects of these agents and a comprehensive understanding of their effects at the molecular level, with the exact signaling pathways and consequences for the whole organism (in the long term) still to be deciphered. Deciphering the mechanisms responsible for the transport of miRs into the mitochondria will, we believe, enable the knowledge gained to be used to develop designs for the transport of drugs/mitomiRs into cells/mitochondria with low toxicity. A detailed understanding of the polyfunctionality/versatility of individual mito-miRs in specific cells (cell cultures, tissues: adipocytes, brain cells) will allow targeting of cellular metabolism to comprehensively block the central link in disease pathogenesis, with low potential side effects of this treatment. At this stage of scientific development, there is not yet a complete picture of the functional potential (target potential) for specific miRs in MetS, the presence of which would avoid the likely toxic effects of miRs/mitomiRs-based therapy [348]. In the review, we have attempted to identify the most important miRs/mitomiRs associated with MetS for mitochondrial function. In our opinion, further research should focus specifically on the miR/mitomiRs described here (Table **1**) and further investigate their potential in the development of MetS and its components. In addition, it is important to pay special attention to the already known mitomiRs and their role in MetS in further practical work, including miR-3, miR-4, miR-15a, miR-25, miR-34b-5p, miR-92a, miR-151a, miR-296-3p, miR-324, miR-378, miR-454-3p, miR-762, miR-1974, miR-1977, miR-1978, miR-2392, miR-5787 [115, 119, 163, 164]. The field of mitochondria-targeted medicine is therefore attracting great interest, and efforts are being made to produce effective nanomedicines. However, there are still a number of unresolved issues related to the exact cell- or tissue-specific characteristics of miR transfer from the cytoplasm to the mitochondria and the utilization of these mechanisms to create new effective targeted constructs against metabolic diseases.

## Figures and Tables

**Table 1:** microRNAs that enter the mitochondrial matrix and regulate mitochondrial biogenesis in the pathogenesis of MetS (and its components).

**miR**	**mitomiR**	**Target Genes**	**Tissue/Cells**	**Expression in MetS/Obesity**	**References**
miR-210	+	NDUFA4, SDHD, COX10, ISCU2	Peripheral blood, resident macrophages, placenta	high	[146, 151, 152, 349]
miR-210-3р	+	COX IV	Mesenchymal stem cells of adipose tissue	high	[176, 175]
miR-210-5р	+	ISCU2, SDH	Bone marrow mesenchymal stem cells	high	[146, 148, 149]
miR-338-5р		COX IV	Hepatocytes (mice)	low	[156]
miR-181с	+	COX1, Sp1, MICU1	Cardiomyocytes (mice)	high	[158]
miR-1	+	COX1	Myocytes (mice)/ Hepatocytes (mice)	low/ high	[350, 351]
miR-378	+	COX1	Hepatocytes (mice)	high	[352]
miR-196а	+	COX IV	Mesenchymal stem cells of adipose tissue	high	[176, 175]
miR-let-7c	+	COX IV	Mesenchymal stem cells of adipose tissue	high	[176, 175]
miR-101-3р	+	COX1	Cardiomyocytes/ blood	high/ low	[353, 354]
miR-762		ND2, COX1, AGAP-2, RNase7, ST2, Rab5a	Adipose tissue macrophages	low	[178]
miR- Let-7b	+	COX genes	Hepatocytes	high	[182]
			Blood, placenta	high	[183]
miR-26а		PDHX, Fbxl19, ADAM17	Hepatocytes (mice, people)	low	[187]
miR-128-3р		PDK1	Hepatocytes	low	[190]
		PPARy	Hepatocytes (mice)	high	[191]
miR-128		PGC-1a, NDUFA4, SDHD	Adipose tissue (mice)	high	[273]
miR-494-3р		CS	3T3-L1 cell cultures, adipocytes, hepatocytes (mice)	high	[192-194]
miR-124		SUCLG2, FOXQ1	Blood	low	[196]
miR-34а	+	DRP1, FGF19, FGF21, Sirt1, PINK1, NDUFS8, COX5b, ATP5a1	Lenticular epitheliocytes, adipocytes, mononuclear cells of adipose tissue, hepatocytes (mice)	high	[220] [203] [219] [208, 215]
miR-21	+	DRP1	Adipose tissue, hepatocytes, blood	high	[163, 228-231]
miR-181а	+	DRP1, BDNF, Bcl-2	Endotheliocytes	high	[242]
miR-181b	+	DRP1, BDNF, PTEN	Blood	high	[205]
			Endothelial cells of adipose tissue (mice)	low	[243]
miR-30а		DRP1, р53, PPARy, ADIPOQ, FABP4, STAT1	Adipose tissue	low	[249, 250]
miR-499-5р		DRP1, PTEN, PRDM16, UCP1, PGC1α, Fnip1	Hepatocytes	high	[253]
miR-761		MFF	Myocytes	high	[258]
miR-200а		MFF	Liver, blood	high	[267, 268]
miR-484		FIS1, SMAD7, YAP1, BCL2L13, Sorbs2	Blood	high	[272]
miR-92а-3р	+	Mfn1, SMAD, KLF2	Blood	high	[292-294]

## LIST OF ABBREVIATIONS

miRs: microRNAs
CerS: Ceramide Synthase
PKCs: Protein Kinase Cs
DAGs: Diacylglycerols
mitomiRs: mitomicroRNAs
ETC: Electron Transport Chain
MetS: Metabolic Syndrome
IR: Insulin Resistance
MSCs: Mesenchymal Stem Cells
